# Cardiac Amyloidosis: A Contemporary Review of Medical and Surgical Therapy

**DOI:** 10.2174/011573403X240302230925043500

**Published:** 2024-10-04

**Authors:** Drew Brownell, Aiswarya J. Pillai, Nandini Nair

**Affiliations:** 1 Division of Cardiology, Department of Medicine, Texas Tech Health Science Center, Lubbock, TX, 79430, USA

**Keywords:** Cardiac amyloidosis, mechanical circulatory support, ATTR, cardiac transplantation, cardiomyopathy

## Abstract

Amyloidosis is a systemic disease initiated by deposition of misfolded proteins in the extracellular space, due to which multiple organs may be affected concomitantly. Cardiac amyloidosis, however, remains a major cause of morbidity and mortality in this population due to infiltrative /restrictive cardiomyopathy. This review attempts to focus on contemporary medical and surgical therapies for the different types of cardiac amyloidosis. Amyloidosis affecting the heart are predominantly of the transthyretin type (acquired in the older or genetic in the younger patients), and the monoclonal immunoglobulin light chain (AL) type which is solely acquired. A rare form of secondary amyloidosis AA type can also affect the heart due to excessive production and accumulation of the acute-phase protein called Serum Amyloid A” (SAA) in the setting of chronic inflammation, cancers or autoinflammatory disease. More commonly AA amyloidosis is seen in the liver and kidney. Other rare types are Apo A1 and Isolated Atrial Amyloidosis (AANF). Medical therapies have made important strides in the clinical management of the two common types of cardiac amyloidosis. Surgical therapies such as mechanical circulatory support and cardiac transplantation should be considered in appropriate patients. Future research using AI driven algorithms for early diagnosis and treatment as well as development of newer genetic engineering technologies will drive improvements in diagnosis, treatment and patient outcomes.

## 1 INTRODUCTION

Amyloidosis is a systemic disease initiated by deposition of misfolded proteins in the extracellular space [1, 2]. Cardiac amyloidosis, however, remains a major cause of morbidity and mortality in this population due to infiltrative /restrictive cardiomyopathy [3-6]. Multiple organs may be affected concomitantly. Amyloidosis affecting the heart are predominantly of the transthyretin type (acquired in the older or genetic in the younger patients), and the monoclonal immunoglobulin light chain (AL) type which is solely acquired [5]. A rare form of secondary amyloidosis AA type can also affect the heart due to excessive production and accumulation of the acute-phase protein called Serum Amyloid A” (SAA) in the setting of chronic inflammation, cancers or autoinflammatory disease. Cardiac involvement is rare with predominance in the kidneys though reports of accelerated stenosis of the aortic and mitral valves have been reported [6]. More commonly AA amyloidosis is seen in the liver and kidney. AA amyloidosis is less common in the United States than in Europe. A hereditary component including gene polymorphisms for the SAA1 isotype also contribute to predisposition to this type in the setting of chronic inflammatory diseases such as the Familial Mediterranean Fever [4-7].

Though other types are rarely found in the cardiac setting it is important to note that greater than 30 proteins can assemble into amyloid fibrils. One such type is a rare hereditary type AAPO A-I cardiac amyloidosis due to mutations in the *AAPOA1* gene. Interestingly, only mutations beyond residue 90 onward lead to cardiac disease. AApo A-I is the second commonest cause of “hereditary” amyloidosis and may be associated with low levels of high-density lipoprotein [8]. Another very rare form of cardiac amyloidosis is Isolated Atrial Amyloidosis (AANF) due to deposition of atrial natriuretic peptide. This peptide is secreted with increased atrial wall stretch. AANF increases with age mostly seen in the ninth decade of life and has a predisposition for the female gender. It occurs in young patients with chronic atrial fibrillation secondary to valvular disease [9]. AANF appears as thin, linear deposits along and underneath the endocardium. Definitive clinical significance is unclear. Lastly, hemodialysis related amyloidosis results in minimal deposition of beta 2 microglobulin in the cardiac tissues but has a predominant involvement of the joints [10-12]. This review attempts to focus on contemporary medical and surgical therapies available for cardiac amyloidosis 

## 2 METHODS

A literature review was conducted from 2000 to 2022 using Google Scholar and PubMed to include papers that address the pathophysiology and treatment of cardiac amyloidosis. The focus of this review is limited to the cardiovascular manifestations of amyloidosis hence papers with more emphasis on systemic amyloidosis involving other organs were excluded.

### 2.1 Medical Management 

Medical management discussed here involves more recently approved treatments and some therapies in different stages of research and development. Medical therapies have made important strides in the clinical management of the two common types of cardiac amyloidosis. Table **1** summarizes selected studies that show the impact of medical management on survival in this population.

### 2.2 Senile Systemic Amyloidosis / Wild Type Transthyretin Amyloidosis (wtATTR)/ Hereditary Amyloidosis (mATTR)

Until recently, medical management for wtATTR and mATTR was limited to supportive therapy and rarely organ transplants. In recent years, specific drugs have been developed that target different elements of amyloid formation and deposition. The primary categories include TTR stabilizers, TTR silencers, and amyloid fibril disruptors. Many of these therapies have been approved for polyneuropathy patients, but are still undergoing clinical trials for use in ATTR cardiac amyloidosis [5, 13].

### 2.3 TTR Stabilizers 

Drugs that stabilize TTR are tafamidis, diflunisal, AG10, green tea extract Epigallocatechin-3-gallate (EGCG), and tolcapone. Most of these work by stabilizing the tetrameric form, preventing dissociation into monomers and subsequent amyloid fibril formation [12, 13].

Tafamidis phase 3 trials showed a reduction in all-cause mortality from 42.9% to 29.5%, and decreased cardiac hospitalizations . It is currently approved by the FDA for ATTR cardiac amyloidosis. The mechanism of action involves binding and stabilization of the TTR tetrameric form, reducing its dissociation into monomers, resulting in the reduction of amyloid fibril formation and deposition [12-15]. Elliott *et al.* showed a reduction in mortality of 44.9% (tafamidis) *vs.* 62.7 (placebo) *p<*0.001 HR =0.59 (Table **1**) [15].

Diflunisal has primarily been used in ATTR polyneuropathy patients. Diflunisal in cardiac amyloidosis patients has been tested in small retrospective studies. In the existing literature 2 studies compared diflunisal *versus* no treatment which showed improvements in cardiac structure and function as deduced by reduced left atrial volume index, improved global longitudinal strain and lower troponin levels. Diflunisal is also associated with decreased mortality and reduced number of orthotopic heart transplants in the transthyretin amyloidosis cardiomyopathy (ATTR-CM) population [16-20]. Diflunisal is associated with gastrointestinal and renal side effects, as well as hypertension and worsening heart failure, so close monitoring is recommended [4,13-18]. In 2 independent studies, diflunisal showed a better survival that was statistically significant (Table **1**) [17, 20].

AG10 is a selective, oral TTR stabilizer for ATTR-CM that mimics the beneficial TTR mutation (Thr119Met). The T119M variant stabilizes TTR mutation to prevent disease. Phase II results indicate almost complete stabilization of transthyretin and elevated serum TTR levels. Treatments with AG10 have been well tolerated. Phase III trials are ongoing [12, 13, 17-20]. At 2 years, improvement in TTR stabilization was noted (Table **1**) [19]. 

Epigallocatechin-3-gallate (EGCG), the green tea polyphenol has been evaluated for its ability to prevent/ delay amyloidosis. EGCG is known to inhibit the aggregation of α-synuclein, amyloid-β, and huntingtin proteins. It has been shown to prevent the formation of fibrils both *in vitro* and *in vivo*, reduce amyloid cytotoxicity, and remodel fibrils to form non-toxic amorphous substances that cannot form toxic fibrils. EGCG acts as an antioxidant but it can itself remain in an oxidized state to promote the remodeling of fibrils *via* the formation of Schiff bases and crosslinking. Microparticles synthesized from oxidized EGCG and loaded with a second anti-amyloidogenic molecule demonstrated a synergistic therapeutic effect. EGCG can prevent amyloid formation and disaggregate amyloid fibrils with no documented serious adverse effects. Two small observational studies have shown inhibition of disease progression, as well as reduction in left ventricular size [4, 12, 18, 21,22,]. No survival benefit was noted (Table **1**) [21]. 

Tolcapone, a therapeutic agent for Parkinson’s disease, has shown strong TTR stabilization in phase II trials. Tolcapone has a potential adverse reaction of acute fulminant liver failure, so its use in ATTR cardiac amyloidosis is rare and should be closely monitored [23-27]. In a rare and fatal form of ATTR called leptomeningeal amyloidosis where TTR accumulates in the brain due to destabilizing TTR mutations, tolcapone may be beneficial because it binds with high affinity /specificity to leptomeningeal TTR variants, stabilizing them and therefore preventing them from aggregating in the brain [23, 27]. No long-term studies have been conducted to assess its survival benefit if any (Table **1**) [23].

TTR tetramer dissociation appears to be rate limiting. Therefore, reducing the rate of dissociation using such kinetic stabilizers would help reduce the rate of progression of cardiomyopathy. Efficacy of each stabilizer to inhibit TTR dissociation is determined by the ratio of the stabilizer's dissociation constants from TTR and albumin because albumin competes with TTR to bind the stabilizers. Additionally, stabilizers with equal potency safety, pharmacokinetics, and distribution in the tissues tissue would influence their use in the clinical setting [24-30].

### 2.4 TTR Silencers 

TTR silencers include patisiran, revusiran, vutrisiran, inotersen, and ION 682884 (AKCEA-TTR-LRx). They are silencing RNAs (SiRNAs) that help to regulate target gene transcription.

### 2.5 SiRNAs

Patisiran is an siRNA that reduces the amount of transcribed TTR. Treatment with patisiran has been associated with a reduction in left ventricular wall thickness, NT-proBNP levels, cardiac hospitalizations, and all-cause mortality in comparison to the placebo group in a phase III trial (Table **1**) [4, 12, 13,18, 31- 33]. Revusiran another siRNA was used in a phase III trial which was stopped due to a higher number of patient deaths in the revusiran arm as compared to the placebo arm (18 (12.9%) *versus* 2 (3%). The cause of deaths were not well defined and most of the deaths were in patients >75 years with advanced heart failure (Table **1**) [12, 34]. Vutrisiran another siRNA is currently in phase III trials to compare its efficacy with that of patisiran (Table **1**) [13,21,25-28,35]. Vutisiran improved all outcomes (Neuropathy Impairment Score, and Quality of life) with acceptable safety and no deaths reported (Table **1**) [35].

### 2.6 Antisense Oligonucleotides

Another molecular approach has been using antisense oligonucleotides. Inotersen is an antisense oligonucleotide that binds to a specific mRNA sequence decreasing pathogenic protein translation. Phase II trials have demonstrated stabilization of left ventricular thickness and mass and good 6-minute walk test results. No major side effects were noted, except for glomerulonephritis and thrombocytopenia rarely seen when inotersen is used for ATTR polyneuropathy [12, 13]. Benson *et al*. showed that inotersen causes less worsening and disease progression with better quality of life in patients with polyneuropathy (Table **1**) [36]. Eplontersen (ION 682884 /AKCEA-TTR-LRx) is another antisense oligonucleotide currently in phase III trials to understand its long-term safety and efficacy in hereditary ATTR with polyneuropathy [12, 13, 22].

### 2.7 TTR Amyloid Fibril Disruptors/Extractors 

These are novel therapeutics that target aberrant proteins, or already formed amyloid fibrils. These include PRX004, NI006, and doxycycline plus tauroursodeoxycholic acid [4, 12, 13, 22].

PRX004 is an anti-TTR antibody that targets misfolded transthyretin and prevents fibril formation. This drug is currently in phase I trials [4, 12, 13, 22]. NI006, another monoclonal antibody, is in phase I/II trials to determine safety and efficacy in inhibiting and clearing ATTR amyloid deposits [12, 22].

A small phase II study of doxycycline plus tauroursodeoxycholic acid showed cardiac disease stabilization during 12 months of treatment [4, 13, 33].

Organ transplantation has become less common among ATTR cardiac amyloidosis patients due to emerging therapies, and possible disease recurrence. However, in lower-risk patients without comorbidities, heart and liver transplants might be effective, especially in mATTR cases. [5,12,13,29].

### 2.8 Light Chain Amyloidosis (AL)

Most treatment options involve reducing free light chain concentration, normalization of serum and urine monoclonal protein levels, and eradication of plasma cells producing aberrant immunoglobulin (4,29). Treatments for AL are primarily derived from multiple myeloma treatment (4). Existing therapy typically involves the combination of melphalan, bortezomib, or stem cell transplant (SCT). Melphalan, an alkylating agent, is used alongside SCT in low-risk patients. In higher-risk patients, or if SCT is not a possibility, melphalan can be used with the proteasome inhibitor bortezomib. The treatment regimen often includes cyclophosphamide and dexamethasone with bortezomib (CyBorD)(4,29,30). Drugs in development either target the plasma cells, or the aberrant protein and amyloid fibrils (29). At 2 years Bortezomib induction showed lower relapse/progression [13(9-18)% *vs.* 23(16-32)%, *p* 0.0^2^], longer progression-free survival (PFS) [82(77-87)% *vs.* 69(61-77)%, *p* <0.0^1^]. Overall survival [92(88-95)% *vs.* 89(84-94)%, *p* 0.^22^], was similar with /without induction (Table **1**) [37].

Daratumumab, an immunotherapy drug targeting plasma cells, has had positive results with minimal side effects. Recent phase III data indicate an improved response rate when given with CyBorD, as compared to CyBorD by itself [4,12]. In a retrospective study, Kennedy *et al*. showed that 1 year survival was 76% in the group treated with daratumumab (Table **1**) [38]. 

In another retrospective study conducted in China Cyclophosphamide, thalidomide, and dexamethasone (CTD) *versus* bortezomib and dexamethasone (Bdex) showed that 1and 2 year overall survival was 90.2% and 81.7% with CTD, and 87.6% and 82.7% with BDex (Table **1**) [39]. Effect noted in Chinese patients may shed light on race -based therapeutic responses. 

A second-generation proteasome inhibitor carfilzomib in phase I/II trials is effective but has serious cardiac, renal, and pulmonary side effects. Carfilzomib use should be limited to patients without heart or kidney deposits [4]. Another second-generation proteasome inhibitor ixazomib has shown promising phase I/II trials with minor side effects. Cardiac toxicity has been noted hence close monitoring should be implemented with the ongoing Phase III trials [4, 12].

Immunomodulators such as thalidomide, as well as thalidomide analogs such as lenalidomide and pomalidomide, can decrease plasma cell proliferation. Thalidomide has been used for multiple myeloma therapy and lenalidomide and pomalidomide have gone through phase II trials. These immunomodulators have shown positive results for AL amyloidosis but can be associated with worsening cardiac symptoms including atrial fibrillation, hypotension, and heart failure. Typically, treatment with immunomodulators is only in refractory AL disease, and if there is resistance to proteasome inhibitors [1, 4]. 

In a retrospective analysis, doxycycline added to standard chemotherapy was associated with cardiac improvement in about 30-40% of patients [4].

-NEOD001, immunotherapy antibody targeting amyloid deposits, had good preliminary results, trials were stopped in 2018 because endpoints were not obtained [1].

Anti-serum amyloid P (miridesap or CPHPC), an antibody that lowers the amyloid p component is present in most amyloid fibrils. This antibody showed promising serum amyloid p depletion in early trials. However, later phase II trials showed little cardiac response hence trials were discontinued in 2018 for risk/benefit reasons [4,12, 40-42]. 

Heart transplant may be considered in some patients. In order to prevent disease recurrence and improve outcomes, heart transplant should be followed by a melphalan plus stem cell transplant [5]. 

### 2.9 Heavy Chain Amyloidosis (AH) /Heavy and Light Chain Amyloidosis (AHL)

AH and AHL are extremely rare forms of amyloidosis. Treatment choices and responses to treatment are similar to those of AL. CyBorD therapy may be useful but side effects of peripheral neuropathy and myelosuppression limit its long-term use [33]. Treatment with lenalidomide and dexamethasone was unsuccessful while daratumumab plus dexamethasone has met with success. Another approach was to use cyclophosphamide and granulocyte colony-stimulating factor, followed by conditioning with melphalan, and a stem cell transplant. 1 year post-transplant proteinuria reduction and no clonal plasma cells were reported in a case report. These studies show promise but need further investigation in larger study populations [43]. 

### 2.10 Serum Amyloid A Amyloidosis / Secondary Systemic Amyloidosis (AA)

The current therapeutic goal for AA amyloidosis is to normalize the concentrations of serum amyloid A and prevent long-term elevated levels. AA is a secondary systemic amyloidosis and is often treated by targeting the underlying etiology. Many different conditions can lead to AA including infectious, chronic inflammatory, autoimmune, and malignant diseases, which are treated by antibiotics, cytokine blockers, immunosuppressants, and chemotherapy respectively [44-47]. Eprodisate was under phase II/III trials to treat patients without any underlying condition. Eprodisate binds to serum amyloid A and was designed to prevent amyloid formation, however, no significant benefit was observed [44-47]. Miridesap (CPHPC), a drug that targets serum amyloid *P* shows decreased amyloid deposits but needs further long-term studies [44]. Other therapeutic agents in development that target the amyloid protein itself, include the monoclonal antibody tocilizumab [45]. However, no therapy specific to AA cardiac involvement has been developed.

### 2.11 Apolipoprotein A-1 Amyloidosis (ApoA1)

ApoA1 systemic amyloidosis is a very rare type of amyloidosis [48]. There are currently no approved therapies, but early trials with miridesap (CPHPC) hope to show decreased levels of serum amyloid P and reduced amyloid buildup in a case report [48]. Treatment is otherwise limited to supportive therapy tailored to the involved organs [48]. Liver transplantation and transplants of other infiltrated organs are effective in patients with ApoA1, and are more successful than transplants for AA and AL amyloidosis. There is recurrence in about 20% of transplant recipients [48].

### 2.12 Isolated Atrial Amyloidosis (AANF)

No specific treatments are currently available for Isolated Atrial Amyloidosis except medications to control the arrhythmias such as atrial fibrillation, which are very common in this population [49, 50]. Calcium channel blockers and digoxin should be avoided for rate control [49]. Control of risk factors such as hypertension, hypertrophy, diabetes, and myocardial ischemia, along with rate/rhythm control, may help to reduce aggregation of aberrant ANP [49].

### 2.13 Supportive Medical Therapy and the Role of GDMT (Guideline Directed Medical Therapy)

Supportive therapy for heart failure, arrythmias, thrombus formation and other cardiac pathologies is similar for all amyloid types that infiltrate the heart [41]. Typical treatment for heart failure is a loop diuretic plus a mineralocorticoid receptor antagonist like spironolactone [12, 13, 22, 41]. Bumetanide and torsemide are recommended due to higher bioavailability [13]. In AL and mATTR subtype patients, diuretics should be closely monitored to avoid worsened orthostatic hypotension (12,13,51). Midodrine may be used to support diuresis [13]. Angiotensin Converting Enzyme inhibitors and Angiotensin II receptor blockers are contraindicated as they can worsen hypotension due to autonomic dysfunction [12,13,22,41]. Beta-blockers are also rarely used as they can decrease cardiac output and cause hypotension in cardiac amyloidosis patients. This is because these patients rely heavily on heart rate due to impaired stroke volume [12,13,22]. In fact, the intolerance of beta blockers is often helpful in reaching a cardiac amyloidosis diagnosis [12,41]. However, in rate management beta blockers may be used with caution and at low dosages [41]. Beta blockers that have alpha-blocking activity such as carvedilol should also be avoided as worsened hypotension may ensue [13]. Calcium channel blockers are detrimental because they bind to amyloid aggregates and precipitate, and have a negative inotropic effect [12,13,22]. Digoxin also binds to amyloid and can be toxic, but recent data suggest that it may still be given at low doses if closely monitored [12]. Arrythmias are much more common in cardiac amyloidosis patients, especially atrial fibrillation, and can be controlled with amiodarone [12,13,22]. Thrombus formation can occur in the atria of cardiac amyloidosis patients, especially AL, and warrants the use of anticoagulants [12,13,22]. Pacemakers have proven helpful for symptomatic relief in patients, but have no impact on patient survival [12,41,42]. Implanted Cardioverter-Defibrillator use has also been studied. No survival benefit has been noted hence it is not recommended for routine use [12,41].

ACE inhibitors (ACEi), angiotensin receptor blockers (ARBs) and mineralocorticoid receptor antagonists (MRAs) may be used in cardiac amyloidosis and up-titrated slowly with no significant safety concerns. Beta-blockers are not well tolerated in AL amyloidosis and may lead to worsening of the hemodynamic profile [51, 52].

## 3 SURGICAL THERAPIES 

### 3.1 Mechanical Circulatory Support

Mechanical circulatory support should be considered in patients with larger left ventricular cavities that will accommodate the cannulas without any arrhythmias and /or suction events. Biventricular failure can be another challenge as these patients have a high risk for right ventricular failure post Left ventricular assist device (LVAD) insertion. Additionally, patients on concomitant chemotherapy or immunotherapy would be predisposed to increased risk of infections. In a single-center study mean survival was noted as 536 days with significantly improved survival in patients with a left ventricular internal dimension >4.6 cms suggesting that larger LV size is an important factor in survival [53].

Biventricular support may be more appropriate in this population. In a single center experience, 9 carefully selected patients underwent transplantation and survival without complications in one year with 2 patients dying on the transplant list [54]. Analysis from the INTERMACS database showed poor survival in patients with cardiac amyloidosis on mechanical support as a bridge to transplantation as compared to patients with dilated cardiomyopathy (DCM) or nonamyloid Restrictive Cardiomyopathy (RCM). Increased rates of complications such as gastrointestinal bleeding, strokes and renal dysfunction were noted in the amyloid population [55].

### 3.2 Organ Transplantation

In the past heart transplantation was contraindicated essentially due to the high mortality. However, present-day improvements in the diagnosis and treatment of ATTR and AL amyloidosis have paved the way for improved outcomes post-cardiac transplantation. Several studies have now shown similar outcomes in the amyloid *versus* non-amyloid patients [56-64]. With the change in organ allocation in 2018 there has been a reduction in mortality on the waiting list and better transplantation rates which makes it more feasible now [61].

The ATTR patients with the wild type are usually older and will not qualify for heart transplantation. However, the mutant types especially those with combined cardiomyopathy and polyneuropathy would need a heart /liver dual organ transplant. In the current times with effective therapies for ATTR, a reduced requirement for double organ transplant could be envisioned. Tailoring of therapies for the different mutations V30M, T60A should be done effectively to prevent unnecessary dual organ transplant and post-transplant complications. 

AL amyloidosis can occur in multiple organs. Therefore, extensive pretransplant evaluation should be done to exclude extracardiac involvement. The interaction of plasma cell–directed therapy and immunosuppression with post-transplantation is relevant but still not clear. Ongoing chemotherapy would be expected to raise the risk of infection. Stem cell transplants seem to increase mortality as reported by some centers [57]. Treatments directed at the reduction of light chains such as the use of bortezomib can decrease rejection risk but those directed at decreasing plasma cells can be associated with increased rejection episodes. Therapies that target plasma cells should be avoided or used with caution in the post-transplant population as they have been associated with increases in rejection episodes [64]. 

## CONCLUSIONS

This review briefly discusses the role of currently available therapies for the different subtypes of amyloidosis. Cardiac amyloidosis is a major cause of morbidity and mortality due to its infiltrative/restrictive nature. Medical therapies have made important strides which have significantly influenced outcomes. Surgical therapies such as mechanical circulatory support and /or cardiac transplantation are viable therapies in some patients to prolong life with good quality. Fig. (**1**) shows a brief summary of medical and surgical therapeutic strategies for cardiac amyloidosis. 

In recent years, many drugs have been developed that target different elements of amyloid formation and deposition for the ATTR subtypes. The drugs include TTR stabilizers, TTR silencers, and amyloid fibril disruptors in different stages of clinical development. For the light chain subtype a combination of cyclophosphamide and dexamethasone with bortezomib (CyBorD) is used. A combination of immunotherapy and CyBorD seems to produce better results. All the drugs in development target the plasma cells or the aberrant protein and amyloid fibrils. A heart transplant can be an option in some patients. However, prevention of recurrence requires treatment with melphalan and stem cell transplant in this population. Stem cell transplant tends to enhance infections and have been reported to increase mortality. Other rare types of amyloidosis such as ApoA1 amyloidosis, AH, AHL, AA and AANF have treatment options that include organ transplantation and treating individual etiologies.

Future research using AI-driven algorithms for early diagnosis and treatment as well as the development of newer genetic engineering technologies such as *in vivo* gene editing will drive improvements in diagnosis, treatment and patient outcomes.

## FUTURE PERSPECTIVES

In the future, standardized protocols should be evolved for the treatment of cardiac amyloidosis which would include appropriate medical therapies coupled with surgical therapies where relevant. The effective treatments post-transplantation will improve outcomes without jeopardizing the immunosuppression required for graft survival.

The use of artificial intelligence (AI) algorithms for early diagnosis and risk stratification will improve outcomes. Such AI-driven algorithms for prediction would be useful for population screening. Therefore, early intervention can improve clinical management and improve the socio-economic impact on the patient and the healthcare system. Such AI-driven algorithms would help physicians in improving healthcare outcomes at a reasonable cost [65-68].

Gene-editing technologies can improve treatment strategies based on molecular mechanisms. Applying such techniques to improve outcomes would constitute an important aspect of future therapies [69].

Current RNA targeting therapies are limited by the long term administration of the drug with considerable side effects. One of the potentially evolving strategies is *in vivo* gene editing [69-73]. In ATTR, a typical monogenic disease process, using short palindromic repeats and associated Cas9 endonuclease (CRISPR-Cas9) system to accomplish *in vivo* gene editing appears to be an exceptionally viable option [69]. In a small clinical trial, a lipid nanoparticle delivery system with liver tropism and target showed a substantial improvement in symptoms in patients with hereditary ATTR amyloidosis and polyneuropathy. The clinical effect was substantiated by decreasing TTR mRNA levels [69]. Extensive testing for the potential for off-target effects of such gene editing should be pursued to prevent unwanted side effects. Using AI-driven modeling to identify nonspecific targets can make the technique of *in vivo* gene editing more specific and reduce/eliminate any unwanted side effects. 

## Figures and Tables

**Fig. (1):**
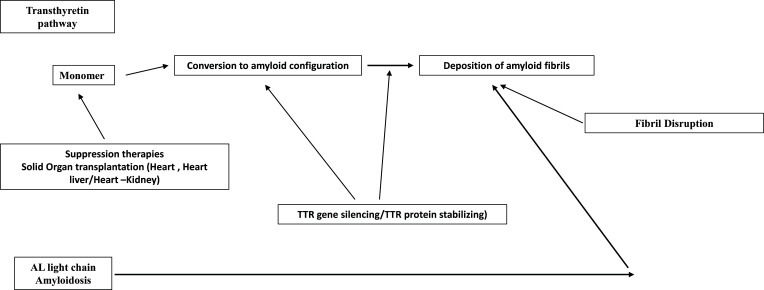
Therapies that target the different stages of amyloid fibril formation and deposition.

**Table 1:** Selected studies addressing survival in amyloidosis patients using existing drugs.

**Study**	**Study Type**	**n**	**Type of Amyloidosis**	**Drug**	**Outcomes**	**Duration of Follow up**	**Conclusions**	**References**
Elliott *et al.*	Phase 3 study data from RCT	331	Wt ATTR/mutant ATTR	Tafamidis	Reduction in mortality 44.9% (tafamidis) *vs.* 62.7 (placebo) *p<*0.001 HR =0.59	72 months	Best survival benefits noted with early diagnosis and treatment.	[15]
-	-	-	-	-	-	-	However treatment at an advanced stage still provides survival benefit	-
Siddiqui *et al.*	Retrospective Cohort Study	104	ATTR wt/mutant	Diflusinal	Reduction in mortality control *versus* diflusinal (p0.0013) HR=0.13	-	Survival curves show 84% reduction in mortality after 4 years	[17]
Hanson *et al.*	Retrospective study	35	wt ATTR	Diflusinal	4 year observed survival in patients with higher levels of stabilized TTR molecules	72 months	TTR concentration >18 mg /ml was associated with improved survival	[20]
Judge *et al.*	Randomized control study ( dose range)	49	ATTR wt/mutant	AG 10	Improvement in TTR stabilization	2 years	Improvement in TTR stabilization	[19]
Cappelli *et al.*	Retrospective study	65	cardiac ATTR	Epigallocatechin-3-gallate (EGCG)	No survival benefit (60 ± 15% *vs*. 61 ± 12%, *p =* 0.276)	691 days	EGCG halts amyloid deposition but has no impact on survival	[21]
Gamez *et al.*	Poof of concept Phase Iia trail	17	Wt ATTR/mutant ATTR	Tolcapone	Acute study to show that tolcapone was able to stabilize TTR	24 hours	No survival study yet on this compound	[23]
-	-	-	-	-	-	-	Increases TTR stabilization by 52 % in 2 hours	-
Solomon *et al.*	RCT phase 3 study	126	hATTR	Patisiran	Cardiac hospitalizations / all-cause death were 18.7 and 10.1 / 100 patient-years	18.7 months (median)	The drug halt or reverse progression	[31]
-	-	-	-	-	(placebo versus patisiran ) hazard ratio, 0.54; 95% CI, 0.28-1.01).	-	-	-
Antonopoulos *et al.*	Meta -analysis	95	ATTR	Tafamidis/Patisiran	2-year survival was better on tafamidis/patisiran *versus* no treatment	pooled 2 yr survival	Treatment with tafamidis/patisiran is better than no treatment	[32]
-	-	-	-	-	(79.9%, 95% CI 74.4-85.3 *vs*. 72.4%, 95% CI 69.8-74.9, *p* < 0.05).	-	-	-
Judge *et al.*	Phase 3 multicenter study	206	hATTR	Revusiran	Eighteen (12.9%) patients on revusiran and 2 (3.0%) on placebo died	stopped after 6.71 months (median)	Stopped due to increased mortality in the treatment arm patients	[34]
-	-	-	-	-	-	-	Heart failure was noted but cause of death was not well defined	-
Adams *et al.*	Phase 3 RCT	241	hTTR -mediated polyneuropathy	Vutisiran	Vutisiran improved all outcomes (Neuropathy Impairment Score, and Quality of life) with acceptable safety	18 months	No deaths reported related to the drug	[35]
Benson *et al.*	Phase 3 RCT	172	ATTRm polyneuropathy	Inotersen	Less worsening and disease progression with better quality of life	66 weeks	Quality of life improved with less disease progression in the inotersen group	[36]
-	-	-	-	-	-	-	Survival not addressed but 5 patients died in inotersen group due to glomerulonephritis	-
-	-	-	-	-	-	-	Grade 4 thrombocytopenia was responsible for 1 death	-
-	-	-	-	-	-	-	No patients died in placebo group	-
Cornell *et al.*	Retrospective Study	440	AL amyloidosis	Pre-transplant Borttezomib induction	Bortezomib induction showed had lower relapse/progression [13(9–18)% *vs.* 23(16–32)%, *p*=0.02].	2 years	Borteozimib induction improved disease progression -free survival at 2 years	[37]
-	-	-	-	Autologous Hematopoietic Cell transplant	Induction showed longer progression-free survival (PFS) [82(77–87)% *vs.* 69(61–77)%, *p* <0.01]	-	-	-
-	-	-	-	irrespective of the plasma cell burden	Overall survival [92(88–95)% *vs.* 89(84–94)%, *p*=0.22], which was similar with /without induction	-	-	-
Kennedy *et al.*	Retrospective Study	21	AL amyloidosis	Daratumumab	67% patients showed a cardiac response	3 years	1 year survival was 76 % in the treated group	[38]
-	-	-	-	-	78% showed a renal response.	-	-	-
Liu *et al.*	Retrospective Study	81	AL amyloidosis	Cyclophosphamide, thalidomide, and dexamethasone (CTD)	One- and 2-year overall survival was 90.2% and 81.7% with CTD, and 87.6% and 82.7% with BDex.	4 years	Effect noted in Chinese patients mat shed light on race -based therapeutic responses	[39]

## LIST OF ABBREVIATIONS

AI: Artificial Intelligence
ARBs: Angiotensin Receptor Blockers
DCM: Dilated Cardiomyopathy
LVAD: Left Ventricular Assist Device
MRAs: Mineralocorticoid Receptor Antagonists
RCM: Restrictive Cardiomyopathy
SCT: Stem Cell Transplant
